# Exacerbation of Fragile X-associated Tremor/Ataxia Syndrome in the Context of COVID-19 Infection: A Case Report

**DOI:** 10.7759/cureus.69969

**Published:** 2024-09-23

**Authors:** Hussein Ghasham, Heather Heild, Rebecca Patel

**Affiliations:** 1 Physical Medicine and Rehabilitation, Touro University Nevada, College of Osteopathic Medicine, Las Vegas, USA; 2 Physical Medicine and Rehabilitation, HCA Healthcare, Las Vegas, USA

**Keywords:** cerebellar ataxia, covid-19, fmr1 gene, fragile x-associated tremor/ataxia syndrome, fxtas (fragile x-associated tremor/ataxia syndrome), neurodegenerative disorder

## Abstract

Fragile X-associated tremor/ataxia syndrome (FXTAS) is a late-onset neurodegenerative disorder affecting premutation carriers of the FMR1 gene. This case report presents a 65-year-old male who was diagnosed with FXTAS after presenting with an acute exacerbation of neurological symptoms in the setting of a COVID-19 infection. The case highlights the potential for viral infections to trigger the worsening of FXTAS symptoms and emphasizes the importance of comprehensive evaluation in such scenarios.

## Introduction

Fragile X-associated tremor/ataxia syndrome (FXTAS) affects approximately one in 4000 men over the age of 55 years in the general population, with about 40% of male premutation carriers developing the syndrome [1]. The core features include intention tremor and cerebellar ataxia, typically beginning after age 60 [1]. This case report describes an unusual presentation of FXTAS exacerbation coinciding with COVID-19 infection, contributing to the limited literature on how viral infections may impact the course of this rare neurodegenerative disorder. FXTAS typically affects older adults, with men being more frequently and severely affected than women due to the lack of a protective second X chromosome. Other signs and symptoms of FXTAS include parkinsonism, cognitive decline, peripheral neuropathy, autonomic dysfunction, and neuropsychiatric symptoms. Diagnosis is usually based on clinical presentation, family history, and characteristic MRI findings, while treatment focuses on symptom management as there is currently no cure.

## Case presentation

A 65-year-old male at a large hospital in the state of Nevada presented to the emergency department with complaints of generalized weakness and lower extremity weakness that began earlier that day. The patient reported an inability to stand or walk independently, a significant change from his baseline ambulatory status with a four-wheel walker. His past medical history was significant for chronic back pain and a previously diagnosed pinched nerve. Notably, in 2012, the patient had a brain MRI after presenting to the ER for dizziness and syncope. Results at that time showed moderate, bilateral, symmetric middle cerebellar peduncle regions of signal hypersensitivity. Differentials per the radiologist report included small vessel ischemia, inflammatory process, demyelinating process, or vasculitis. However, before a neurologist could be consulted the patient was discharged home and the patient was lost to follow up.

On initial evaluation, the patient was found to be febrile and tested positive for COVID-19. Despite the positive test, the patient was asymptomatic for COVID-19 except for the weakness in his legs. Neurological examination revealed generalized weakness without signs of focal weakness, sensory changes, aphasia, cranial nerve deficits, or upper motor neuron signs. CT of the head without contrast showed marked hypodensity in the bilateral cerebellar white matter, which increased compared to a prior study from 2012. Lumbar CT revealed mild to moderate degenerative changes in the mid to lower lumbar spine with areas of the central canal and neural foraminal stenosis.

**Figure 1:**
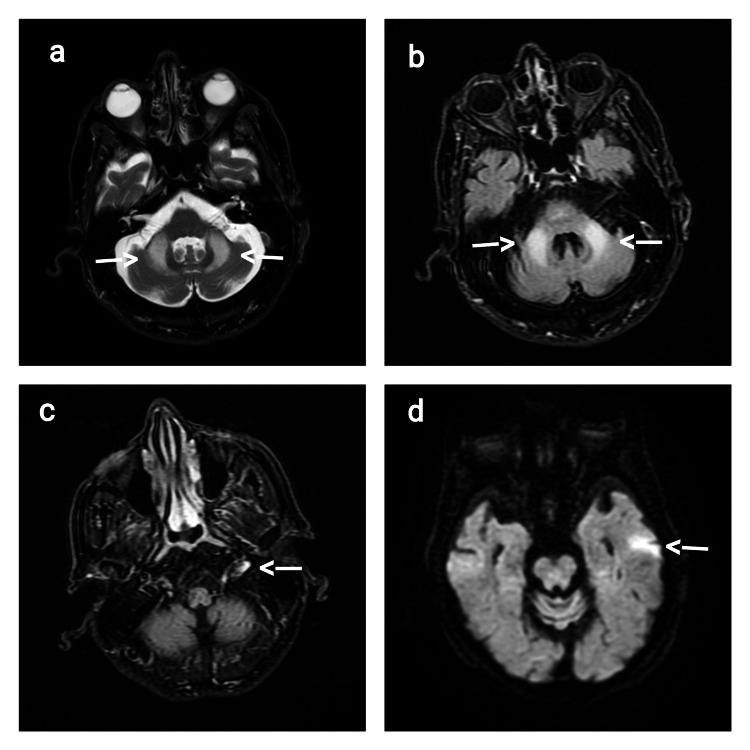
Brain magnetic resonance imaging (2024). (a, b) MRI of the brain showing an interval increase in the extent of previously seen bilateral middle cerebellar peduncle and cerebellar hemispheric foci of increased signal on DWI with no signal drop on ADC. (c, d) MRI of the brain showing new scattered foci of increased signal observed along the splenium of the corpus callosum and the left glossopharyngeal nerve. DWI: diffusion-weighted imaging; ADC: apparent diffusion coefficient.

MRI of the brain and spine was completed and demonstrated an interval increase in the extent of previously seen bilateral middle cerebellar peduncle and cerebellar hemispheric foci of increased signal on diffusion-weighted imaging (DWI) with no signal drop on apparent diffusion coefficient (ADC) (Figure 1). New scattered foci of increased signal were observed along the splenium of the corpus callosum and the left glossopharyngeal nerve. These findings were consistent with leukoencephalopathy in the cerebellum, characteristic of FXTAS [2], but raised the possibility of an exacerbation of the underlying condition.

Upon discussing the imaging findings with the patient, he revealed that two of his grandchildren from his daughter have a history of fragile X syndrome, indicating a familial link. This information, combined with the imaging findings, led to the diagnosis of FXTAS. Further assessment using standardized screening tools provided additional insights into the patient's condition.

The Scale for the Assessment and Rating of Ataxia (SARA) was used to quantify the severity of ataxia, focusing on gait, stance, speech, and limb coordination [3]. A score of 8 indicates mild ataxia, suggesting minimal dependence in daily activities. This helps the healthcare team gauge the impact on the patient’s quality of life and tailor interventions accordingly.

The Patient Health Questionnaire-9 (PHQ-9) is a widely-used screening tool for depression, assessing symptoms like mood, energy, and interest in activities [4]. A score of 3 suggests minimal depressive symptoms, indicating that depression is not significantly affecting the patient’s current mental health.

The Montreal Cognitive Assessment (MoCA) evaluates cognitive functions, including memory, attention, language, and executive function [5]. A score of 22 suggests mild cognitive impairment, prompting the healthcare team to monitor and manage potential cognitive decline.

A lumbar puncture was performed, and CSF analysis results showed colorless fluid with <0.002 RBCs, <0.003 WBCs, glucose at 70 mg/dl, total protein at 34 mg/dl, nonreactive venereal disease research laboratory (VDRL), and negative fungal antibodies. This helped to rule out other acute neurological processes. The patient was transferred to the rehab unit and through rigorous physical and occupational therapy, the patient's weakness gradually improved over the course of his hospital stay, approaching his baseline functional status.

## Discussion

This case presents several noteworthy aspects. The acute exacerbation of FXTAS symptoms coinciding with COVID-19 infection suggests a potential link between viral infections and the worsening of neurological symptoms in FXTAS patients [1]. The MRI findings of increased signal abnormalities in characteristic locations for FXTAS (middle cerebellar peduncles, cerebellar hemispheres, and corpus callosum) during the acute presentation highlight the dynamic nature of the disease process [6]. The involvement of the left glossopharyngeal nerve, an unusual finding in FXTAS, raises questions about the full spectrum of cranial nerve involvement in this syndrome. The patient's gradual return to baseline with supportive care underscores the potential for reversibility of acute exacerbations in FXTAS.

Similar to most cases of FXTAS, patients gradually presented with ataxia, especially with gait over a few weeks late in age. In comparison to past cases, the involvement of the glossopharyngeal nerve is a unique finding. Previous studies have primarily focused on the middle cerebellar peduncle sign and cerebellar atrophy. Although many patients can present with neuropsychiatric symptoms [7], our patient’s low score on the PHQ-9 and lack of other psychiatric symptoms did not suggest some changes. This patient also had asymptomatic COVID-19, which exacerbated his symptoms to the point he could not walk and required acute rehab. This is the first recorded case of FXTAS exacerbation with viral infections. This case adds to the vast variability of cognitive, psychiatric, and ataxic symptoms a patient with FXTAS can present with. This case underscores the importance of considering FXTAS in patients with a family history of fragile X syndrome and atypical neurological presentations.

## Conclusions

This case report illustrates the potential for COVID-19 infection to trigger an exacerbation of neurological symptoms in patients with FXTAS. It highlights the importance of comprehensive neurological evaluation, including advanced imaging, in FXTAS patients presenting with acute changes in functional status. Further research is needed to elucidate the relationship between viral infections and FXTAS progression, which could inform management strategies for this patient population.
